# Dysregulated proteostasis network in neuronal diseases

**DOI:** 10.3389/fcell.2023.1075215

**Published:** 2023-02-24

**Authors:** Ching-San Tseng, Yu-Wen Chao, Yi-Hsiang Liu, Yi-Shuian Huang, Hsu-Wen Chao

**Affiliations:** ^1^ Department of Anatomy, School of Medicine, China Medical University, Taichung, Taiwan; ^2^ Department of Physiology, School of Medicine, College of Medicine, Taipei Medical University, Taipei, Taiwan; ^3^ Graduate Institute of Medical Sciences, College of Medicine, Taipei Medical University, Taipei, Taiwan; ^4^ Institute of Biomedical Sciences, Academia Sinica, Taipei, Taiwan; ^5^ Department of Biomedical Science and Environmental Biology, Kaohsiung Medical University, Kaohsiung, Taiwan

**Keywords:** protein degradation, mRNA translation, stress granule, neurodegeneration, post-translation modification

## Abstract

Long-term maintenance of synaptic connections is important for brain function, which depends on varying proteostatic regulations to govern the functional integrity of neuronal proteomes. Proteostasis supports an interconnection of pathways that regulates the fate of proteins from synthesis to degradation. Defects in proteostatic signaling are associated with age-related functional decline and neurodegenerative diseases. Recent studies have advanced our knowledge of how cells have evolved distinct mechanisms to safely control protein homeostasis during synthesis, folding and degradation, and in different subcellular organelles and compartments. Neurodegeneration occurs when these protein quality controls are compromised by accumulated pathogenic proteins or aging to an irreversible state. Consequently, several therapeutic strategies, such as targeting the unfolded protein response and autophagy pathways, have been developed to reduce the burden of misfolded proteins and proved useful in animal models. Here, we present a brief overview of the molecular mechanisms involved in maintaining proteostatic networks, along with some examples linking dysregulated proteostasis to neuronal diseases.

## Introduction

From day-to-day operations to adapting to environmental stress, biological processes in living cells are accomplished through the spatiotemporal and dynamic integration of protein networks, known as proteostasis, which is composed of various molecular processes, including protein synthesis, folding, modification, delivery, and degradation. Neurons are non-dividing and highly polarized cells with specialized and extended compartments, such as axons and dendrites, for transmitting directional information between different circuits through specialized structures called synapses. To support synaptic connectivity and communication over time, tight control of protein synthesis and degradation is required to shape the synaptic proteome for the long-term maintenance of synaptic structure and function. Once neuronal proteostasis declines due to aging or extreme stress, proteome integrity may fail to be preserved, and the resulting misfolded proteins ultimately lead to neuronal disorders (Hetz, 2021). Here, we summarize the current understanding and recent findings regarding the mechanistic and functional roles of proteostatic pathways in neurons.

### Imbalanced mRNA translation in neuronal disorders

**FIGURE 1:**
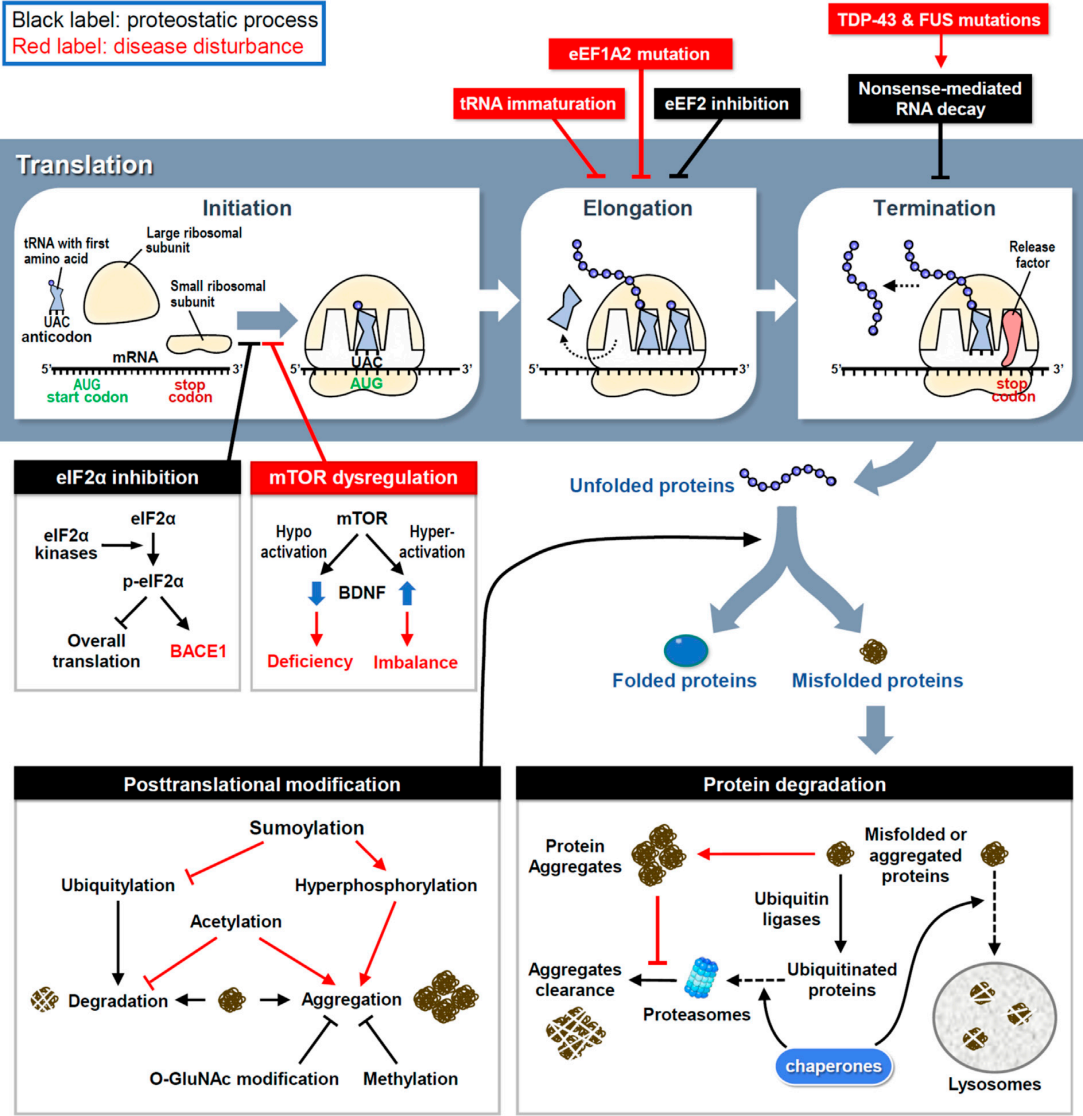
The proteostasis network and disease disturbances. RNA translation is divided into three steps and controlled by proteostatic processes. Under cellular stress, the assembly of initiation and elongation complexes is inhibited to reduce protein synthesis. Moreover, the premature transcript is degraded by NMD to avoid the generation of aberrant proteins. Mutations in key factors involved in translational procedures induce proteome stress and cause neuronal disorders. Following synthesis, nascent proteins experience posttranslational modifications to achieve their correct folding and become functional. However, misfolded proteins are generated by proteome stress and eliminated by three types of protein degradation mechanisms. Under neuropathies, improper modifications and resistances to degradation attenuate the clearance of misfolded proteins and finally cause the protein aggregation.

Ribosomes are where the mRNA is translated into protein products (Figure 1). Upon activation, eukaryotic initiation factor 2 (eIF2) first forms a ternary complex (TC) with the initiator methionyl tRNA and 40S small ribosome, which scans along the mRNA to localize the start codon AUG and triggers the initiation of translation (Cao et al., 2019). Four members of eIF2α kinases in response to various cellular stresses can phosphorylate eIF2α to reduce TC formation and global translation (Bhattarai et al., 2020); however, activating transcription 4 (ATF4) mRNA is preferentially translated by a mechanism involving upstream open reading frames (Zhou et al., 2018). Consequently, stressed cells retain most of their energy to synthesize essential and stress-responsive proteins for survival (Advani and Ivanov, 2019). Unfortunately, some of the induced proteins, including beta-site amyloid precursor protein cleaving enzyme-1 (BACE1), may increase the production of pathological proteins in neurodegenerative diseases, such as β-amyloid precipitation in Alzheimer’s disease (AD) (O'Connor et al., 2008). Another major pathway controlling translation initiation depends on the activity of a protein kinase called mammalian target of rapamycin (mTOR), which phosphorylates eIF4E and its binding proteins to enhance cap-dependent translation initiation (Weiss et al., 2021). Dysregulation of mTOR activity disrupts the balanced production of newly synthesized proteins (Figure 1). Hyperactivated mTOR signaling in Down’s syndrome mouse neurons augments dendritic translation and brain-derived neurotrophic factor (BDNF) synthesis, resulting in insensitivity to extracellular BDNF signaling (Troca-Marin et al., 2011). Conversely, hypoactivated mTOR signaling in Rett’s syndrome downregulates BDNF expression and impairs synaptic connections (Pejhan et al., 2020).

After initiation, eukaryotic elongation factors (eEFs) cooperate to decode the open reading frame sequences and control the rate of protein synthesis. Dysfunction of the elongation complex may lead to neuronal diseases, such as the early stage of epilepsy and intellectual disability with mutations in eEF1A2 (Long et al., 2020). To decelerate translation under cellular stress aggregated pathological proteins, eEF2 kinase (eEF2K) phosphorylates eEF2 to inhibit eEF2-mediated translocation of nascent peptidyl-tRNAs on ribosomes (Fabbri et al., 2021). Indeed, elevated eEF2K activity has been identified in postmortem brains of patients with AD and Parkinson’s disease (PD) (Jan et al., 2017; Jan et al., 2018). However, the excessive elongation brake attenuates neuronal responses to oxidative stress (Jan et al., 2017; Jan et al., 2018), so genetic or pharmacological inhibition of eEF2K can rescue neuronal loss and behavioral defects in mouse models of AD, PD and Dravet syndrome (Beretta et al., 2022). Mutations in the highly conserved elongator complex for tRNA modification impair tRNA maturation and the level of charged aminoacyl-tRNA, thereby leading to imbalanced protein synthesis and familial dysautonomia, a rare genetic disorder of the autonomic nervous system (Lefler et al., 2015; Chaverra et al., 2017).

Before termination, transcripts harboring a premature stop codon are eliminated to avoid the synthesis of aberrant proteins *via* a mechanism known as non-sense-mediated RNA decay (NMD) (Kurosaki et al., 2019). In amyotrophic lateral sclerosis (ALS) and frontotemporal dementia (FTD), mutations in transactive response DNA-binding protein 43 (TDP-43) and fused in sarcoma (FUS) cause aberrant RNA splicing, and some of the mis-spliced transcripts need to be degraded through NMD; thus, enhancing NMD activity protects neurons in the cellular models of ALS and FTD (Barmada et al., 2015). Moreover, oxidative stress induces the cleavage of 3′-untranslated region (UTR) of RNA and consequently the accumulation of isolated 3′-UTRs in ribosomes generates redundant short-peptides and hinders translation (Sudmant et al., 2018). Several polymorphisms in the 3′-UTRs of disease-associated genes have been identified (Grunblatt et al., 2019); however, whether they contribute to isolated 3′-UTR-related pathogenesis requires further investigations.

### Defective post-translational modifications in neuropathology

Some nascent proteins undergo covalent and enzymatic modifications, known as post-translational modification (PTM), to achieve correct folding (Figure 1). Aberrant PTMs can produce misfolded proteins; if not eliminated properly, they can increase the probability of forming aggregates, which are toxic components in many neurodegenerative diseases. For example, hyperphosphorylation of tau enhances the formation of tau tangles, a pathological hallmark of AD (Bai et al., 2020). More than one-third of amino acids in tau can be post-translationally modified, of which approximately one-fifth are phosphorylated (Alquezar et al., 2020). In response to aggregation, ubiquitylation of tau triggers its degradation by the ubiquitin-proteasome system (UPS) (see the next section for details). Methylation of lysine residues also attenuates tau aggregation during aging and AD progression (Balmik and Chinnathambi, 2021). Although methylation can compete with ubiquitylation to modify some lysine residues in tau with the same potency to inhibit tau aggregation, methylation cannot recruit the UPS to degrade tau (Balmik and Chinnathambi, 2021). Moreover, O-linked β-N-acetylglucosamine (O-GlcNAc) modification of tau has been found in AD patients, suggesting that proteostatic signaling is designed to counteract tau phosphorylation and aggregation to slow disease progression (Wang et al., 2017; Wang et al., 2020). In contrast, tau acetylation prevents the degradation of phosphorylated tau and promotes its aggregation (Caballero et al., 2021). Another PTM that reciprocally opposes ubiquitylation is sumoylation, which induces tau hyperphosphorylation and inhibits its degradation (Luo et al., 2014). The location and frequency of PTMs on tau change over time as tauopathy progresses, revealing tau-associated molecular signatures at distinct disease stages (Wesseling et al., 2020). Collectively, these studies demonstrate that inappropriate PTMs impair neuronal proteostasis to drive neuropathogenesis.

### Impaired degradation of protein aggregates in neurodegenerative diseases

The accumulation of specific protein aggregates is a hallmark of many neurodegenerative diseases, so the clearance of neurotoxic aggregates is a challenging task for neurons. To restore proteostasis, misfolded and aggregated proteins must be eliminated by chaperone, UPS or lysosome (Figure 1). Unfortunately, this refolding/degradation machinery is compromised by aggregated proteins that cause neurodegeneration. UPS-dependent protein degradation is achieved by enzymatic addition of polyubiquitin chains to target proteins, followed by their recruitment to the 26S proteasome. Conjugated ubiquitins have been detected in extracellular Aβ plaques in AD brains (Bellia et al., 2019), implying the failure of intracellular protein degradation. Specifically, as extracellular Aβ peptides enter neurons, proteasomal activity is decreased in the cortical regions of patients with AD (Keck et al., 2003; Oh et al., 2005). Furthermore, intracellular aggregation of tau in AD, α-synuclein in PD, and huntingtin in Huntington's disease (HD) also impairs proteasomal activity to enhance neuropathies (Liu et al., 2019; Suzuki et al., 2020; Franco-Iborra et al., 2021).

The lysosome receives cytoplasmic content for enzymatic degradation in a highly conserved catabolic process known as autophagy. Lysosome biogenesis is primarily controlled by transcription factor EB (TFEB), which activates the transcription of genes responsible for lysosome formation and autophagy induction (Kobayashi et al., 2019). TFEB downregulation and abnormal autophagy have been reported in patients with AD and ALS (Tiribuzi et al., 2014; Wang et al., 2016b), and genetic or pharmacological induction of TFEB attenuates neuronal loss and pathological features in AD and PD mouse models (Wang et al., 2016a; Zhuang et al., 2020). Moreover, mutations in other autophagy-associated genes have been shown to accelerate the neuropathogenesis of ALS, FTD, PD and microcephaly (Tresse et al., 2010; Garcia-Sanz et al., 2017; Deng et al., 2020; Almannai et al., 2022). Furthermore, mutant tau and mutant α-synuclein exhibit strong resistance to autophagy (Caballero et al., 2018; Kirchner et al., 2019), commensurate with the difficulty in removing these neurotoxic aggregates.

Three major subtypes of autophagy have been identified in mammalian cells: macroautophagy, microautophagy, and chaperone-mediated autophagy (CMA) (Yim and Mizushima, 2020). In contrast to microautophagy and CMA, which directly transport small amounts of cytosolic materials to lysosomes, macroautophagy begins with the *de novo* synthesis of double-membraned vesicles known as autophagosomes, which sequester large cargos, including damaged organelles (Andrejeva et al., 2020; Schutter et al., 2020). In neurons, most autophagosomes are generated in the distal axon and then transported retrogradely toward the somatic and dendritic compartments (Maday and Holzbaur, 2014). Dystrophic and swollen neurites with accumulated autophagosomes are typical and correlated with synaptic dysfunction in the early stage of AD progression (Sharoar et al., 2019). Additionally, excessive autophagosomes have also been observed in cellular models of PD and ALS (Dehay et al., 2010; Morselli et al., 2011). Although the autophagosome formation is impervious to HD pathology, aggregated autophagosomes and impaired autophagy have been also observed in mouse and cell models of HD (Martinez-Vicente et al., 2010). Notably, depletion of wild-type huntingtin results in the abnormal accumulation of defective autophagosomes because of its function in mediating retrograde transport of autophagosomes along the axon (Zheng et al., 2010). Genetic or pharmacological activation of autophagy attenuates pathological protein aggregation and reduces neuronal pathology in animal models of neurodegenerative diseases (Heckmann et al., 2019; Pupyshev et al., 2019; Brattas et al., 2021; Xu et al., 2022). In addition to promoting translation, mTOR signaling plays an essential role in hindering autophagy induction. Therefore, pharmacological inhibition of mTOR enhances autophagy to remove protein aggregates and ameliorate neurodegeneration (Casillas-Espinosa et al., 2020).

In addition to assisting the conformational folding of proteins, chaperones deliver misfolded proteins to proteasomes and lysosomes for degradation. Chaperones help ubiquitin ligases recognize misfolded targets (Ciechanover and Kwon, 2017) and bring misfolded proteins to lysosomal membranes for embedding (Johnston and Samant, 2021). Importantly, chaperones can disengage insoluble proteins from stable aggregates that are believed to be further refolded or degraded (Shorter, 2011). During aging or neurodegeneration, the balance of chaperone expression is temporarily altered: some chaperones are induced to defend against proteostatic stress, while others are reduced due to disease insults (Auzmendi-Iriarte and Matheu, 2020). Although insufficient degradation of aggregated proteins eventually disrupts proteostasis and causes neuronal death, it also implies potential therapeutics for treating neurodegenerative diseases. For example, the genetic induction of chaperone or delivery of chaperone-simulating nanomaterials facilitates the clearance of neurotoxic proteins and promotes neuronal survival (Huang et al., 2014; Ma et al., 2022). Chaperones require non-client-binding partners as regulators of chaperone action. For example, the binding of misfolded proteins to heat shock protein (HSP) 70 is commenced by interaction with its co-chaperone HSP40 (Morgner et al., 2015). The induction of co-chaperones also alleviates the neurotoxicity-caused by pathogenic proteins (Park et al., 2018). Another example is Valosin-containing protein precursor (VCP)/p97, which is a chaperone containing ATPase activity to assist protein folding, sorting or degradation (Parzych et al., 2019). By ATP hydrolysis-dependent changes of its conformation, VCP/p97 interacts with more than 30 cofactors that connect it to different targets (Riehl et al., 2021). Dysfunction of VCP/p97 impacts various cellular activities and mutations in VCP/p97 are associated with several neurological disorders including ALS (Hall et al., 2017; Matsubara et al., 2021).

### Disturbance of organelle proteostasis in neuronal diseases

**FIGURE 2:**
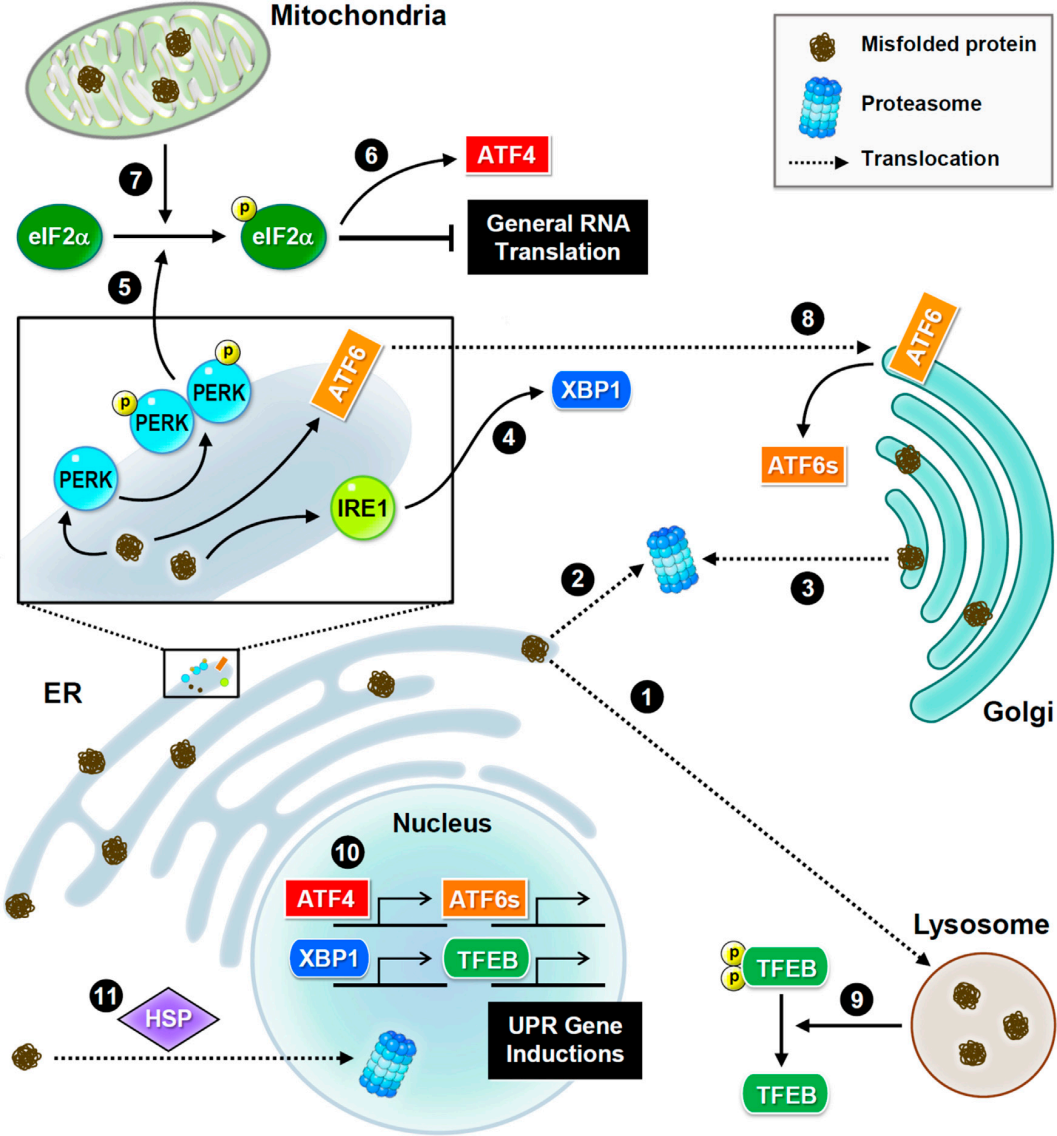
Subcellular proteostasis and UPR signaling. Schematic presentations of proteostasis signaling and protein trafficking in distinct organelles. Main stress sensors and transcription factors are presented. Misfolded proteins are transferred between the ER and Golgi apparatus for refolding, or to the lysosome or nucleus for degradation. In the ER, three major UPR signaling pathways trigger proteostatic processes. First, PERK phosphorylates eIF2α to inhibit the assembly of the initiation ternary complex, thereby suppressing general translation. Additionally, mitochondrial damage also induces phosphorylation of eIF2α. Conversely, some proteins such as transcription factor ATF4 are preferentially produced. Second, IRE1 mediates RNA splicing and promotes the synthesis of transcription factor XBP1. Third, ATF6 is transferred to the Golgi apparatus and cleaved into a short and active form. Furthermore, the proteome stress dephosphorylates and activates transcription factor TFEB. Together, these transcription factors drive the induction of UPR genes to ensure proteostasis.

In living cells, protein quality control requires the cooperation of not only lysosomes but also other organelles. Membrane and secretory proteins are synthesized by ribosomes on the endoplasmic reticulum (ER), and then enter the ER tubules for a series of PTMs to complete conformational folding. Critically, misfolded proteins are detected and transferred to the endolysosomal or proteasomal system for degradation. Other organelles, including the nucleus, Golgi apparatus, and mitochondria, also collaborate to maintain proteostasis through specific and partially overlapping molecular pathways (Figure 2). Errors in protein synthesis cause prolonged expression of misfolded proteins to induce ER stress and the unfolded protein response (UPR), and consequently activates a conserved proteostatic pathway known as ER-associated protein degradation (ERAD) (Gariballa and Ali, 2020). During ERAD, a cascade of enzymatic ubiquitination processes labels misfolded protein substrates and directs them to proteasomal degradation (Carroll and Marqusee, 2022). When the ERAD capacity is overwhelmed by increasing accumulation of pathological proteins, neurons begin to undergo neurodegeneration (Abisambra et al., 2013; Leitman et al., 2013).

UPR signaling is initiated by three transmembrane sensors: protein kinase R-like ER kinase (PERK, which is an eIF2α kinase), inositol-requiring enzyme 1 (IRE1), and activating transcription factor 6 (ATF6) (Figure 2). Genetic or pharmacological manipulation of UPR signaling changes the pathological progression in mouse models of neurodegeneration and aging (Ganz et al., 2020; Krukowski et al., 2020). Upon ER stress, PERK phosphorylates eIF2α, and consequently inhibits translational initiation to relieve the loading of protein synthesis (Bhattarai et al., 2020). Conversely, some transcripts with unique characteristics, such as ATF4, are preferentially translated in response to stress (Zhou et al., 2018). ER stress also degrades mRNAs *via* the endonuclease activity of IRE1 to reduce protein synthesis (Tavernier et al., 2017). IRE1 also mediates splicing of X-box binding protein 1 (XBP1) mRNA to produce transcripts for making functional XBP1 proteins, which then translocate to the nucleus to activate transcription of genes involved in UPR and ERAD (Belyy et al., 2020). Following stress-induced translocation to the Golgi apparatus, ATF6 is proteolytically processed to release a cytosolic fragment that then enters the nucleus and activates the transcription of numerous genes involved in protein folding and degradation (Glembotski et al., 2019). In conclusion, selective inductions of UPR genes under global translational repression are caused by proteome stress. Elevated UPR activation is frequently observed in neurodegenerative brains, because the accumulation of misfolded proteins is a hallmark of neurodegeneration (Hughes and Mallucci, 2019).

Similar to the response to ER stress, mitochondrial damage also activates eIF2α kinases to phosphorylate eIF2α (Fessler et al., 2020; Guo et al., 2020) and eventually initiates eIF2α-independent translation of pro-survival factors. Mitochondria also uptake misfolded proteins from the cytoplasmic matrix to activate their own UPR (Ruan et al., 2017). Interestingly, exogenous mitochondria can be delivered from astrocytes to neurons to help neurons recover after injury (Hayakawa et al., 2016). Unfortunately, mitochondrial function and integrity decline in aging and neurodegenerative diseases (Godoy et al., 2021). When mitochondria are irreversibly damaged, they can be removed *via* mitophagy, a subtype of macroautophagy. However, excessive mitophagy hinders neuronal recovery from UPR stress, which is often observed in neurodegenerative diseases (Fang et al., 2019; Yakhine-Diop et al., 2019).

As a post-ER compartment in the synthesis of membrane and secretory proteins, the Golgi apparatus controls protein quality through two distinct pathways, returning abnormal proteins back to the ER (Brauer et al., 2019; Pennauer et al., 2022) or transferring these proteins for lysosomal degradation (Hellerschmied et al., 2019). Continuous sorting of mutant proteins from the Golgi apparatus to the ER leads to aberrant accumulation in neurodegeneration (Sirkis et al., 2017). Interestingly, the population of misfolded mutant prions persists in the Golgi rather than in the ER (Ashok and Hegde, 2009; Zavodszky and Hegde, 2019), implying that mutant prions have altered trafficking routes and/or the resistance to protein quality control. In contrast to the well-studied ER and mitochondrial UPR mechanisms, Golgi UPR is less understood. Accumulating evidence indicates that Golgi stress responses can trigger specific transcriptional signals (Taniguchi et al., 2016; Serebrenik et al., 2018) and activate ER-resident molecular chaperones (Miyata et al., 2013).

Although the nucleus rarely encounters the accumulation of misfolded proteins, it retains proteasome-dependent degradation to ensure its architecture and genome stability (Almacellas et al., 2021; Shmueli et al., 2022). Nuclear proteasomes eliminate not only nuclear proteins but also proteins transported from the cytoplasmic compartment. Under cellular stress, heat shock proteins help deliver misfolded proteins from the cytoplasm to the nucleus for proteasomal degradation (den Brave et al., 2020), suggesting their essential role in partitioning protein degradation loads between the cytoplasmic and nuclear compartments. However, several neuropathic proteins, such as mutant huntingtin and aggregated tau, impede nucleocytoplasmic transport (Grima et al., 2017; Lester et al., 2021), thereby attenuating protein turnover and leading to neurodegeneration.

### Aberrant aggregation of neuropathic proteins in stress granules

In addition to membrane-bound organelles, many membrane-less organelles are liquid-like droplets that arise from the condensation of cellular materials. Membrane-less RNA-containing organelles can exist constantly like nucleoli and P-bodies or form under specific conditions such as stress granules (SGs), all of which contribute to proteostatic regulation, including ribosome biogenesis, RNA degradation, and translational repression (Riggs et al., 2020; Lafontaine et al., 2021). The formation of these membrane-less condensates depends on the sequestration of biomolecules, including RNAs, RNA-binding proteins, and other proteins, which function like liquid droplets that allow the molecular components to switch between diluted and condensed phases (Espinosa et al., 2020). This demixing phenomenon is referred to as liquid-liquid phase separation (LLPS). By locally increasing the protein concentration, these granules create a condition for phase separation between dissolution and accumulation of internal proteins, finally leading to protein condensation with solid-like characteristics (Guo et al., 2018; Garaizar et al., 2022). Interestingly, many RNA-binding proteins contain not only RNA-binding domains, but also intrinsically disordered regions that drive phase transitions to assemble RNA granules that include translationally silenced mRNAs (Hayashi et al., 2021). Stress-induced eIF2α phosphorylation also initiates the transient assembly of SGs containing 40S ribosomal subunits, translation initiation factors, RNA-binding proteins and mRNAs, thereby retaining these molecules for protein synthesis after recovery from stress (Riggs et al., 2020).

Under prolonged cellular stress, phase separation can also promote the formation of insoluble protein aggregates. The assembly of SGs is initially beneficial because the high concentration of RNA and poly ADP-ribose (PAR) keeps proteins accumulated during liquid-liquid phase separation (McGurk et al., 2018a; Mann et al., 2019). However, the persistent or repetitive assembly of SGs evolves the phase transition into neurotoxic aggregates (Hofweber et al., 2018; Zhang et al., 2019). Numerous disease-related proteins, including FUS, Tau, and TDP-43, have been reported to aggregate in liquid droplets (Murthy et al., 2019; Conicella et al., 2020; Parolini et al., 2022). FUS and TDP-43 are nucleus-abundant RNA-binding proteins that are phase-segregated into SGs in the cytoplasm; therefore, such a stress-induced phase transition has been proposed to facilitate their cytoplasmic aggregation to cause ALS. Moreover, pathogenic mutations in the diverse regions of FUS and TDP-43, including RNA recognition motifs, oligomerization domains and intrinsically disordered and low-complexity regions, promote phase separation and protein aggregation (McGurk et al., 2018a; Murthy et al., 2019; Conicella et al., 2020) because some mutations of FUS and TDP-43 disrupt their electrostatic interactions through posttranslational modifications such as phosphorylation and subsequently affect protein phase separation (McGurk et al., 2018a; Owen et al., 2020). Furthermore, the genetic or pharmacological inhibition of PARylation suppresses phase separation and granule formation (McGurk et al., 2018b; Duan et al., 2019). During AD progression, tau protein also displays an intrinsically disordered conformation, which can undergo liquid-liquid phase separation and eventually become neurotoxic aggregates (Boyko et al., 2020; Parolini et al., 2022).

## Conclusion

From mRNA translation to protein degradation, the proteostatic machinery ensures the functional and conformational integrity of neuronal proteomes. Many molecular pathways have been discovered to contribute to the proteostatic networks of different organelles. Recently, accumulating evidence has shown that defective protein quality control caused by accumulating pathogenic proteins and the aging-associated decline in the regulation of proteostasis have a dramatic impact on the progression of neurodegenerative diseases. Further research may provide the basis for understanding the neuropathy caused by misfolded and aggregated proteins to facilitate the development of clinical applications.

## References

[B1] Abisambra J. F. Jinwal U. K. Blair L. J. O'Leary J. C. 3rd Li Q. Brady S. (2013). Tau accumulation activates the unfolded protein response by impairing endoplasmic reticulum-associated degradation. J. Neurosci. 33, 9498–9507. 10.1523/JNEUROSCI.5397-12.2013 23719816 PMC3733249

[B2] Advani V. M. Ivanov P. (2019). Translational control under stress: Reshaping the translatome. Bioessays 41, e1900009. 10.1002/bies.201900009 31026340 PMC6541386

[B3] Almacellas E. Pelletier J. Day C. Ambrosio S. Tauler A. Mauvezin C. (2021). Lysosomal degradation ensures accurate chromosomal segregation to prevent chromosomal instability. Autophagy 17, 796–813. 10.1080/15548627.2020.1764727 32573315 PMC8032240

[B4] Almannai M. Marafi D. Abdel-Salam G. M. H. Zaki M. S. Duan R. Calame D. (2022). El-Hattab-Alkuraya syndrome caused by biallelic WDR45B pathogenic variants: Further delineation of the phenotype and genotype. Clin. Genet. 101, 530–540. 10.1111/cge.14132 35322404 PMC9359317

[B5] Alquezar C. Arya S. Kao A. W. (2020). Tau post-translational modifications: Dynamic transformers of tau function, degradation, and aggregation. Front. Neurol. 11, 595532. 10.3389/fneur.2020.595532 33488497 PMC7817643

[B6] Andrejeva G. Gowan S. Lin G. Wong Te Fong A. L. Shamsaei E. Parkes H. G. (2020). De novo phosphatidylcholine synthesis is required for autophagosome membrane formation and maintenance during autophagy. Autophagy 16, 1044–1060. 10.1080/15548627.2019.1659608 31517566 PMC7469489

[B7] Ashok A. Hegde R. S. (2009). Selective processing and metabolism of disease-causing mutant prion proteins. PLoS Pathog. 5, e1000479. 10.1371/journal.ppat.1000479 19543376 PMC2691595

[B8] Auzmendi-Iriarte J. Matheu A. (2020). Impact of chaperone-mediated autophagy in brain aging: Neurodegenerative diseases and glioblastoma. Front. Aging Neurosci. 12, 630743. 10.3389/fnagi.2020.630743 33633561 PMC7901968

[B9] Bai B. Wang X. Li Y. Chen P. C. Yu K. Dey K. K. (2020). Deep multilayer brain proteomics identifies molecular networks in alzheimer's disease progression. Neuron 106, 700. 10.1016/j.neuron.2020.04.031 32437656 PMC7322979

[B10] Balmik A. A. Chinnathambi S. (2021). Methylation as a key regulator of Tau aggregation and neuronal health in Alzheimer's disease. Cell Commun. Signal 19, 51. 10.1186/s12964-021-00732-z 33962636 PMC8103764

[B11] Barmada S. J. Ju S. Arjun A. Batarse A. Archbold H. C. Peisach D. (2015). Amelioration of toxicity in neuronal models of amyotrophic lateral sclerosis by hUPF1. Proc. Natl. Acad. Sci. U. S. A. 112, 7821–7826. 10.1073/pnas.1509744112 26056265 PMC4485101

[B12] Bellia F. Lanza V. Garcia-Vinuales S. Ahmed I. M. M. Pietropaolo A. Iacobucci C. (2019). Ubiquitin binds the amyloid beta peptide and interferes with its clearance pathways. Chem. Sci. 10, 2732–2742. 10.1039/c8sc03394c 30996991 PMC6419943

[B13] Belyy V. Tran N. H. Walter P. (2020). Quantitative microscopy reveals dynamics and fate of clustered IRE1α. Proc. Natl. Acad. Sci. U. S. A. 117, 1533–1542. 10.1073/pnas.1915311117 31871156 PMC6983381

[B14] Beretta S. Gritti L. Ponzoni L. Scalmani P. Mantegazza M. Sala M. (2022). Rescuing epileptic and behavioral alterations in a Dravet syndrome mouse model by inhibiting eukaryotic elongation factor 2 kinase (eEF2K). Mol. Autism 13, 1. 10.1186/s13229-021-00484-0 34980259 PMC8722032

[B15] Bhattarai K. R. Chaudhary M. Kim H. R. Chae H. J. (2020). Endoplasmic reticulum (ER) stress response failure in diseases. Trends Cell Biol. 30, 672–675. 10.1016/j.tcb.2020.05.004 32561138

[B16] Boyko S. Surewicz K. Surewicz W. K. (2020). Regulatory mechanisms of tau protein fibrillation under the conditions of liquid-liquid phase separation. Proc. Natl. Acad. Sci. U. S. A. 117, 31882–31890. 10.1073/pnas.2012460117 33262278 PMC7749306

[B17] Brattas P. L. Hersbach B. A. Madsen S. Petri R. Jakobsson J. Pircs K. (2021). Impact of differential and time-dependent autophagy activation on therapeutic efficacy in a model of Huntington disease. Autophagy 17, 1316–1329. 10.1080/15548627.2020.1760014 32374203 PMC8204969

[B18] Brauer P. Parker J. L. Gerondopoulos A. Zimmermann I. Seeger M. A. Barr F. A. (2019). Structural basis for pH-dependent retrieval of ER proteins from the Golgi by the KDEL receptor. Science 363, 1103–1107. 10.1126/science.aaw2859 30846601 PMC7617890

[B19] Caballero B. Bourdenx M. Luengo E. Diaz A. Sohn P. D. Chen X. (2021). Acetylated tau inhibits chaperone-mediated autophagy and promotes tau pathology propagation in mice. Nat. Commun. 12, 2238. 10.1038/s41467-021-22501-9 33854069 PMC8047017

[B20] Caballero B. Wang Y. Diaz A. Tasset I. Juste Y. R. Stiller B. (2018). Interplay of pathogenic forms of human tau with different autophagic pathways. Aging Cell 17, e12692. 10.1111/acel.12692 29024336 PMC5770880

[B21] Cao Y. Liu S. Liu K. Abbasi I. H. R. Cai C. Yao J. (2019). Molecular mechanisms relating to amino acid regulation of protein synthesis. Nutr. Res. Rev. 32, 183–191. 10.1017/S0954422419000052 31097041

[B22] Carroll E. C. Marqusee S. (2022). Site-specific ubiquitination: Deconstructing the degradation tag. Curr. Opin. Struct. Biol. 73, 102345. 10.1016/j.sbi.2022.102345 35247748 PMC9208700

[B23] Casillas-Espinosa P. M. Ali I. O'Brien T. J. (2020). Neurodegenerative pathways as targets for acquired epilepsy therapy development. Epilepsia Open 5, 138–154. 10.1002/epi4.12386 32524040 PMC7278567

[B24] Chaverra M. George L. Mergy M. Waller H. Kujawa K. Murnion C. (2017). The familial dysautonomia disease gene IKBKAP is required in the developing and adult mouse central nervous system. Dis. Model Mech. 10, 605–618. 10.1242/dmm.028258 28167615 PMC5451171

[B25] Ciechanover A. Kwon Y. T. (2017). Protein quality control by molecular chaperones in neurodegeneration. Front. Neurosci. 11, 185. 10.3389/fnins.2017.00185 28428740 PMC5382173

[B26] Conicella A. E. Dignon G. L. Zerze G. H. Schmidt H. B. D'Ordine A. M. Kim Y. C. (2020). TDP-43 alpha-helical structure tunes liquid-liquid phase separation and function. Proc. Natl. Acad. Sci. U. S. A. 117, 5883–5894. 10.1073/pnas.1912055117 32132204 PMC7084079

[B27] Dehay B. Bove J. Rodriguez-Muela N. Perier C. Recasens A. Boya P. (2010). Pathogenic lysosomal depletion in Parkinson's disease. J. Neurosci. 30, 12535–12544. 10.1523/JNEUROSCI.1920-10.2010 20844148 PMC6633458

[B28] den Brave F. Cairo L. V. Jagadeesan C. Ruger-Herreros C. Mogk A. Bukau B. (2020). Chaperone-mediated protein disaggregation triggers proteolytic clearance of intra-nuclear protein inclusions. Cell Rep. 31, 107680. 10.1016/j.celrep.2020.107680 32492414 PMC7273177

[B29] Deng Z. Lim J. Wang Q. Purtell K. Wu S. Palomo G. M. (2020). ALS-FTLD-linked mutations of SQSTM1/p62 disrupt selective autophagy and NFE2L2/NRF2 anti-oxidative stress pathway. Autophagy 16, 917–931. 10.1080/15548627.2019.1644076 31362587 PMC7144840

[B30] Duan Y. Du A. Gu J. Duan G. Wang C. Gui X. (2019). PARylation regulates stress granule dynamics, phase separation, and neurotoxicity of disease-related RNA-binding proteins. Cell Res. 29, 233–247. 10.1038/s41422-019-0141-z 30728452 PMC6460439

[B31] Espinosa J. R. Joseph J. A. Sanchez-Burgos I. Garaizar A. Frenkel D. Collepardo-Guevara R. (2020). Liquid network connectivity regulates the stability and composition of biomolecular condensates with many components. Proc. Natl. Acad. Sci. U. S. A. 117, 13238–13247. 10.1073/pnas.1917569117 32482873 PMC7306995

[B32] Fabbri L. Chakraborty A. Robert C. Vagner S. (2021). The plasticity of mRNA translation during cancer progression and therapy resistance. Nat. Rev. Cancer 21, 558–577. 10.1038/s41568-021-00380-y 34341537

[B33] Fang E. F. Hou Y. Palikaras K. Adriaanse B. A. Kerr J. S. Yang B. (2019). Mitophagy inhibits amyloid-beta and tau pathology and reverses cognitive deficits in models of Alzheimer's disease. Nat. Neurosci. 22, 401–412. 10.1038/s41593-018-0332-9 30742114 PMC6693625

[B34] Fessler E. Eckl E. M. Schmitt S. Mancilla I. A. Meyer-Bender M. F. Hanf M. (2020). A pathway coordinated by DELE1 relays mitochondrial stress to the cytosol. Nature 579, 433–437. 10.1038/s41586-020-2076-4 32132706 PMC7116715

[B35] Franco-Iborra S. Plaza-Zabala A. Montpeyo M. Sebastian D. Vila M. Martinez-Vicente M. (2021). Mutant HTT (huntingtin) impairs mitophagy in a cellular model of Huntington disease. Autophagy 17, 672–689. 10.1080/15548627.2020.1728096 32093570 PMC8032238

[B36] Ganz J. Shacham T. Kramer M. Shenkman M. Eiger H. Weinberg N. (2020). A novel specific PERK activator reduces toxicity and extends survival in Huntington's disease models. Sci. Rep. 10, 6875. 10.1038/s41598-020-63899-4 32327686 PMC7181660

[B37] Garaizar A. Espinosa J. R. Joseph J. A. Krainer G. Shen Y. Knowles T. P. J. (2022). Aging can transform single-component protein condensates into multiphase architectures. Proc. Natl. Acad. Sci. U. S. A. 119, e2119800119. 10.1073/pnas.2119800119 35727989 PMC9245653

[B38] Garcia-Sanz P. Orgaz L. Bueno-Gil G. Espadas I. Rodriguez-Traver E. Kulisevsky J. (2017). N370S-GBA1 mutation causes lysosomal cholesterol accumulation in Parkinson's disease. Mov. Disord. 32, 1409–1422. 10.1002/mds.27119 28779532

[B39] Gariballa N. Ali B. R. (2020). Endoplasmic reticulum associated protein degradation (ERAD) in the pathology of diseases related to TGFβ signaling pathway: Future therapeutic perspectives. Front. Mol. Biosci. 7, 575608. 10.3389/fmolb.2020.575608 33195419 PMC7658374

[B40] Glembotski C. C. Rosarda J. D. Wiseman R. L. (2019). Proteostasis and beyond: ATF6 in ischemic disease. Trends Mol. Med. 25, 538–550. 10.1016/j.molmed.2019.03.005 31078432 PMC6592750

[B41] Godoy J. A. Rios J. A. Picon-Pages P. Herrera-Fernandez V. Swaby B. Crepin G. (2021). Mitostasis, calcium and free radicals in health, aging and neurodegeneration. Biomolecules 11, 11071012. 10.3390/biom11071012 PMC8301949 34356637

[B42] Grima J. C. Daigle J. G. Arbez N. Cunningham K. C. Zhang K. Ochaba J. (2017). Mutant huntingtin disrupts the nuclear pore complex. Neuron 94, 93–107.e6. 10.1016/j.neuron.2017.03.023 28384479 PMC5595097

[B43] Grunblatt E. Werling A. M. Roth A. Romanos M. Walitza S. (2019). Association study and a systematic meta-analysis of the VNTR polymorphism in the 3'-UTR of dopamine transporter gene and attention-deficit hyperactivity disorder. J. Neural Transm. (Vienna) 126, 517–529. 10.1007/s00702-019-01998-x 30923918 PMC6456487

[B44] Guo L. Kim H. J. Wang H. Monaghan J. Freyermuth F. Sung J. C. (2018). Nuclear-import receptors reverse aberrant phase transitions of RNA-binding proteins with prion-like domains. Cell 173, 677–692.e20. 10.1016/j.cell.2018.03.002 29677512 PMC5911940

[B45] Guo X. Aviles G. Liu Y. Tian R. Unger B. A. Lin Y. T. (2020). Mitochondrial stress is relayed to the cytosol by an OMA1-DELE1-HRI pathway. Nature 579, 427–432. 10.1038/s41586-020-2078-2 32132707 PMC7147832

[B46] Hall C. E. Yao Z. Choi M. Tyzack G. E. Serio A. Luisier R. (2017). Progressive motor neuron pathology and the role of astrocytes in a human stem cell model of VCP-related ALS. Cell Rep. 19, 1739–1749. 10.1016/j.celrep.2017.05.024 28564594 PMC5464993

[B47] Hayakawa K. Esposito E. Wang X. Terasaki Y. Liu Y. Xing C. (2016). Transfer of mitochondria from astrocytes to neurons after stroke. Nature 535, 551–555. 10.1038/nature18928 27466127 PMC4968589

[B48] Hayashi Y. Ford L. K. Fioriti L. McGurk L. Zhang M. (2021). Liquid-liquid phase separation in physiology and pathophysiology of the nervous system. J. Neurosci. 41, 834–844. 10.1523/JNEUROSCI.1656-20.2020 33472825 PMC7880275

[B49] Heckmann B. L. Teubner B. J. W. Tummers B. Boada-Romero E. Harris L. Yang M. (2019). LC3-Associated endocytosis facilitates beta-amyloid clearance and mitigates neurodegeneration in murine alzheimer's disease. Cell 178, 536–551.e14. 10.1016/j.cell.2019.05.056 31257024 PMC6689199

[B50] Hellerschmied D. Serebrenik Y. V. Shao L. Burslem G. M. Crews C. M. (2019). Protein folding state-dependent sorting at the Golgi apparatus. Mol. Biol. Cell 30, 2296–2308. 10.1091/mbc.E19-01-0069 31166830 PMC6743468

[B51] Hetz C. (2021). Adapting the proteostasis capacity to sustain brain healthspan. Cell 184, 1545–1560. 10.1016/j.cell.2021.02.007 33691137

[B52] Hofweber M. Hutten S. Bourgeois B. Spreitzer E. Niedner-Boblenz A. Schifferer M. (2018). Phase separation of FUS is suppressed by its nuclear import receptor and arginine methylation. Cell 173, 706–719.e13. 10.1016/j.cell.2018.03.004 29677514

[B53] Huang F. Wang J. Qu A. Shen L. Liu J. Liu J. (2014). Maintenance of amyloid beta peptide homeostasis by artificial chaperones based on mixed-shell polymeric micelles. Angew. Chem. Int. Ed. Engl. 53, 8985–8990. 10.1002/anie.201400735 24985739

[B54] Hughes D. Mallucci G. R. (2019). The unfolded protein response in neurodegenerative disorders - therapeutic modulation of the PERK pathway. FEBS J. 286, 342–355. 10.1111/febs.14422 29476642

[B55] Jan A. Jansonius B. Delaidelli A. Bhanshali F. An Y. A. Ferreira N. (2018). Activity of translation regulator eukaryotic elongation factor-2 kinase is increased in Parkinson disease brain and its inhibition reduces alpha synuclein toxicity. Acta Neuropathol. Commun. 6, 54. 10.1186/s40478-018-0554-9 29961428 PMC6027557

[B56] Jan A. Jansonius B. Delaidelli A. Somasekharan S. P. Bhanshali F. Vandal M. (2017). eEF2K inhibition blocks Aβ42 neurotoxicity by promoting an NRF2 antioxidant response. Acta Neuropathol. 133, 101–119. 10.1007/s00401-016-1634-1 27752775

[B57] Johnston H. E. Samant R. S. (2021). Alternative systems for misfolded protein clearance: Life beyond the proteasome. FEBS J. 288, 4464–4487. 10.1111/febs.15617 33135311

[B58] Keck S. Nitsch R. Grune T. Ullrich O. (2003). Proteasome inhibition by paired helical filament-tau in brains of patients with Alzheimer's disease. J. Neurochem. 85, 115–122. 10.1046/j.1471-4159.2003.01642.x 12641733

[B59] Kirchner P. Bourdenx M. Madrigal-Matute J. Tiano S. Diaz A. Bartholdy B. A. (2019). Proteome-wide analysis of chaperone-mediated autophagy targeting motifs. PLoS Biol. 17, e3000301. 10.1371/journal.pbio.3000301 31150375 PMC6561683

[B60] Kobayashi T. Piao W. Takamura T. Kori H. Miyachi H. Kitano S. (2019). Enhanced lysosomal degradation maintains the quiescent state of neural stem cells. Nat. Commun. 10, 5446. 10.1038/s41467-019-13203-4 31784517 PMC6884460

[B61] Krukowski K. Nolan A. Frias E. S. Boone M. Ureta G. Grue K. (2020). Small molecule cognitive enhancer reverses age-related memory decline in mice. Elife 9, e62048. 10.7554/eLife.62048 33258451 PMC7721440

[B62] Kurosaki T. Popp M. W. Maquat L. E. (2019). Quality and quantity control of gene expression by nonsense-mediated mRNA decay. Nat. Rev. Mol. Cell Biol. 20, 406–420. 10.1038/s41580-019-0126-2 30992545 PMC6855384

[B63] Lafontaine D. L. J. Riback J. A. Bascetin R. Brangwynne C. P. (2021). The nucleolus as a multiphase liquid condensate. Nat. Rev. Mol. Cell Biol. 22, 165–182. 10.1038/s41580-020-0272-6 32873929

[B64] Lefler S. Cohen M. A. Kantor G. Cheishvili D. Even A. Birger A. (2015). Familial dysautonomia (FD) human embryonic stem cell derived PNS neurons reveal that synaptic vesicular and neuronal transport genes are directly or indirectly affected by IKBKAP downregulation. PLoS One 10, e0138807. 10.1371/journal.pone.0138807 26437462 PMC4593545

[B65] Leitman J. Ulrich Hartl F. Lederkremer G. Z. (2013). Soluble forms of polyQ-expanded huntingtin rather than large aggregates cause endoplasmic reticulum stress. Nat. Commun. 4, 2753. 10.1038/ncomms3753 24217578

[B66] Lester E. Ooi F. K. Bakkar N. Ayers J. Woerman A. L. Wheeler J. (2021). Tau aggregates are RNA-protein assemblies that mislocalize multiple nuclear speckle components. Neuron 109, 1675–1691.e9. 10.1016/j.neuron.2021.03.026 33848474 PMC8141031

[B67] Liu X. Hebron M. L. Mulki S. Wang C. Lekah E. Ferrante D. (2019). Ubiquitin specific protease 13 regulates tau accumulation and clearance in models of alzheimer's disease. J. Alzheimers Dis. 72, 425–441. 10.3233/JAD-190635 31594232

[B68] Long K. Wang H. Song Z. Yin X. Wang Y. (2020). EEF1A2 mutations in epileptic encephalopathy/intellectual disability: Understanding the potential mechanism of phenotypic variation. Epilepsy Behav. 105, 106955. 10.1016/j.yebeh.2020.106955 32062104

[B69] Luo H. B. Xia Y. Y. Shu X. J. Liu Z. C. Feng Y. Liu X. H. (2014). SUMOylation at K340 inhibits tau degradation through deregulating its phosphorylation and ubiquitination. Proc. Natl. Acad. Sci. U. S. A. 111, 16586–16591. 10.1073/pnas.1417548111 25378699 PMC4246270

[B70] Ma X. Lu C. Chen Y. Li S. Ma N. Tao X. (2022). CCT2 is an aggrephagy receptor for clearance of solid protein aggregates. Cell 185, 1325–1345.e22. 10.1016/j.cell.2022.03.005 35366418

[B71] Maday S. Holzbaur E. L. (2014). Autophagosome biogenesis in primary neurons follows an ordered and spatially regulated pathway. Dev. Cell 30, 71–85. 10.1016/j.devcel.2014.06.001 25026034 PMC4109719

[B72] Mann J. R. Gleixner A. M. Mauna J. C. Gomes E. DeChellis-Marks M. R. Needham P. G. (2019). RNA binding antagonizes neurotoxic phase transitions of TDP-43. Neuron 102, 321–338.e8. 10.1016/j.neuron.2019.01.048 30826182 PMC6472983

[B73] Martinez-Vicente M. Talloczy Z. Wong E. Tang G. Koga H. Kaushik S. (2010). Cargo recognition failure is responsible for inefficient autophagy in Huntington's disease. Nat. Neurosci. 13, 567–576. 10.1038/nn.2528 20383138 PMC2860687

[B74] Matsubara T. Izumi Y. Oda M. Takahashi M. Maruyama H. Miyamoto R. (2021). An autopsy report of a familial amyotrophic lateral sclerosis case carrying VCP Arg487His mutation with a unique TDP-43 proteinopathy. Neuropathology 41, 118–126. 10.1111/neup.12710 33415820

[B75] McGurk L. Gomes E. Guo L. Mojsilovic-Petrovic J. Tran V. Kalb R. G. (2018a). Poly(ADP-Ribose) prevents pathological phase separation of TDP-43 by promoting liquid demixing and stress granule localization. Mol. Cell 71, 703–717.e9. 10.1016/j.molcel.2018.07.002 30100264 PMC6128762

[B76] McGurk L. Mojsilovic-Petrovic J. Van Deerlin V. M. Shorter J. Kalb R. G. Lee V. M. (2018b). Nuclear poly(ADP-ribose) activity is a therapeutic target in amyotrophic lateral sclerosis. Acta Neuropathol. Commun. 6, 84. 10.1186/s40478-018-0586-1 30157956 PMC6114235

[B77] Miyata S. Mizuno T. Koyama Y. Katayama T. Tohyama M. (2013). The endoplasmic reticulum-resident chaperone heat shock protein 47 protects the Golgi apparatus from the effects of O-glycosylation inhibition. PLoS One 8, e69732. 10.1371/journal.pone.0069732 23922785 PMC3726774

[B78] Morgner N. Schmidt C. Beilsten-Edmands V. Ebong I. O. Patel N. A. Clerico E. M. (2015). Hsp70 forms antiparallel dimers stabilized by post-translational modifications to position clients for transfer to Hsp90. Cell Rep. 11, 759–769. 10.1016/j.celrep.2015.03.063 25921532 PMC4431665

[B79] Morselli E. Marino G. Bennetzen M. V. Eisenberg T. Megalou E. Schroeder S. (2011). Spermidine and resveratrol induce autophagy by distinct pathways converging on the acetylproteome. J. Cell Biol. 192, 615–629. 10.1083/jcb.201008167 21339330 PMC3044119

[B80] Murthy A. C. Dignon G. L. Kan Y. Zerze G. H. Parekh S. H. Mittal J. (2019). Molecular interactions underlying liquid-liquid phase separation of the FUS low-complexity domain. Nat. Struct. Mol. Biol. 26, 637–648. 10.1038/s41594-019-0250-x 31270472 PMC6613800

[B81] O'Connor T. Sadleir K. R. Maus E. Velliquette R. A. Zhao J. Cole S. L. (2008). Phosphorylation of the translation initiation factor eIF2alpha increases BACE1 levels and promotes amyloidogenesis. Neuron 60, 988–1009. 10.1016/j.neuron.2008.10.047 19109907 PMC2667382

[B82] Oh S. Hong H. S. Hwang E. Sim H. J. Lee W. Shin S. J. (2005). Amyloid peptide attenuates the proteasome activity in neuronal cells. Mech. Ageing Dev. 126, 1292–1299. 10.1016/j.mad.2005.07.006 16153690

[B83] Owen I. Rhoads S. Yee D. Wyne H. Gery K. Hannula I. (2020). The prion-like domain of Fused in Sarcoma is phosphorylated by multiple kinases affecting liquid- and solid-phase transitions. Mol. Biol. Cell 31, 2522–2536. 10.1091/mbc.E20-05-0290 32877292 PMC7851872

[B84] Park S. K. Arslan F. Kanneganti V. Barmada S. J. Purushothaman P. Verma S. C. (2018). Overexpression of a conserved HSP40 chaperone reduces toxicity of several neurodegenerative disease proteins. Prion 12, 16–22. 10.1080/19336896.2017.1423185 29308690 PMC5871033

[B85] Parolini F. Tira R. Barracchia C. G. Munari F. Capaldi S. D'Onofrio M. (2022). Ubiquitination of Alzheimer's-related tau protein affects liquid-liquid phase separation in a site- and cofactor-dependent manner. Int. J. Biol. Macromol. 201, 173–181. 10.1016/j.ijbiomac.2021.12.191 35016968

[B86] Parzych K. Saavedra-Garcia P. Valbuena G. N. Al-Sadah H. A. Robinson M. E. Penfold L. (2019). The coordinated action of VCP/p97 and GCN2 regulates cancer cell metabolism and proteostasis during nutrient limitation. Oncogene 38, 3216–3231. 10.1038/s41388-018-0651-z 30626938 PMC6756015

[B87] Pejhan S. Del Bigio M. R. Rastegar M. (2020). The MeCP2E1/E2-BDNF-miR132 homeostasis regulatory network is region-dependent in the human brain and is impaired in Rett syndrome patients. Front. Cell Dev. Biol. 8, 763. 10.3389/fcell.2020.00763 32974336 PMC7471663

[B88] Pennauer M. Buczak K. Prescianotto-Baschong C. Spiess M. (2022). Shared and specific functions of Arfs 1-5 at the Golgi revealed by systematic knockouts. J. Cell Biol. 221, e202106100. 10.1083/jcb.202106100 34749397 PMC8579194

[B89] Pupyshev A. B. Tikhonova M. A. Akopyan A. A. Tenditnik M. V. Dubrovina N. I. Korolenko T. A. (2019). Therapeutic activation of autophagy by combined treatment with rapamycin and trehalose in a mouse MPTP-induced model of Parkinson's disease. Pharmacol. Biochem. Behav. 177, 1–11. 10.1016/j.pbb.2018.12.005 30582934

[B90] Riehl J. Rijal R. Nitz L. Clemen C. S. Hofmann A. Eichinger L. (2021). Domain organization of the UBX domain containing protein 9 and analysis of its interactions with the homohexameric AAA + ATPase p97 (Valosin-Containing protein). Front. Cell Dev. Biol. 9, 748860. 10.3389/fcell.2021.748860 34631722 PMC8495200

[B91] Riggs C. L. Kedersha N. Ivanov P. Anderson P. (2020). Mammalian stress granules and P bodies at a glance. J. Cell Sci. 133, jcs242487. 10.1242/jcs.242487 32873715 PMC10679417

[B92] Ruan L. Zhou C. Jin E. Kucharavy A. Zhang Y. Wen Z. (2017). Cytosolic proteostasis through importing of misfolded proteins into mitochondria. Nature 543, 443–446. 10.1038/nature21695 28241148 PMC5793917

[B93] Schutter M. Giavalisco P. Brodesser S. Graef M. (2020). Local fatty acid channeling into phospholipid synthesis drives phagophore expansion during autophagy. Cell 180, 135–149.e14. 10.1016/j.cell.2019.12.005 31883797

[B94] Serebrenik Y. V. Hellerschmied D. Toure M. Lopez-Giraldez F. Brookner D. Crews C. M. (2018). Targeted protein unfolding uncovers a Golgi-specific transcriptional stress response. Mol. Biol. Cell 29, 1284–1298. 10.1091/mbc.E17-11-0693 29851555 PMC5994893

[B95] Sharoar M. G. Hu X. Ma X. M. Zhu X. Yan R. (2019). Sequential formation of different layers of dystrophic neurites in Alzheimer's brains. Mol. Psychiatry 24, 1369–1382. 10.1038/s41380-019-0396-2 30899091 PMC7204504

[B96] Shmueli M. D. Sheban D. Eisenberg-Lerner A. Merbl Y. (2022). Histone degradation by the proteasome regulates chromatin and cellular plasticity. FEBS J. 289, 3304–3316. 10.1111/febs.15903 33914417 PMC9292675

[B97] Shorter J. (2011). The mammalian disaggregase machinery: Hsp110 synergizes with Hsp70 and Hsp40 to catalyze protein disaggregation and reactivation in a cell-free system. PLoS One 6, e26319. 10.1371/journal.pone.0026319 22022600 PMC3194798

[B98] Sirkis D. W. Aparicio R. E. Schekman R. (2017). Neurodegeneration-associated mutant TREM2 proteins abortively cycle between the ER and ER-Golgi intermediate compartment. Mol. Biol. Cell 28, 2723–2733. 10.1091/mbc.E17-06-0423 28768830 PMC5620379

[B99] Sudmant P. H. Lee H. Dominguez D. Heiman M. Burge C. B. (2018). Widespread accumulation of ribosome-associated isolated 3' UTRs in neuronal cell populations of the aging brain. Cell Rep. 25, 2447–2456.e4. 10.1016/j.celrep.2018.10.094 30485811 PMC6354779

[B100] Suzuki G. Imura S. Hosokawa M. Katsumata R. Nonaka T. Hisanaga S. I. (2020). α-synuclein strains that cause distinct pathologies differentially inhibit proteasome. Elife 9, e56825. 10.7554/eLife.56825 32697196 PMC7406352

[B101] Taniguchi M. Sasaki-Osugi K. Oku M. Sawaguchi S. Tanakura S. Kawai Y. (2016). MLX is a transcriptional repressor of the mammalian Golgi stress response. Cell Struct. Funct. 41, 93–104. 10.1247/csf.16005 27251850

[B102] Tavernier S. J. Osorio F. Vandersarren L. Vetters J. Vanlangenakker N. Van Isterdael G. (2017). Regulated IRE1-dependent mRNA decay sets the threshold for dendritic cell survival. Nat. Cell Biol. 19, 698–710. 10.1038/ncb3518 28459443 PMC5563826

[B103] Tiribuzi R. Crispoltoni L. Porcellati S. Di Lullo M. Florenzano F. Pirro M. (2014). miR128 up-regulation correlates with impaired amyloid β(1-42) degradation in monocytes from patients with sporadic Alzheimer's disease. Neurobiol. Aging 35, 345–356. 10.1016/j.neurobiolaging.2013.08.003 24064186

[B104] Tresse E. Salomons F. A. Vesa J. Bott L. C. Kimonis V. Yao T. P. (2010). VCP/p97 is essential for maturation of ubiquitin-containing autophagosomes and this function is impaired by mutations that cause IBMPFD. Autophagy 6, 217–227. 10.4161/auto.6.2.11014 20104022 PMC2929010

[B105] Troca-Marin J. A. Alves-Sampaio A. Montesinos M. L. (2011). An increase in basal BDNF provokes hyperactivation of the Akt-mammalian target of rapamycin pathway and deregulation of local dendritic translation in a mouse model of Down's syndrome. J. Neurosci. 31, 9445–9455. 10.1523/JNEUROSCI.0011-11.2011 21715609 PMC6623175

[B106] Wang H. Wang R. Carrera I. Xu S. Lakshmana M. K. (2016a). TFEB overexpression in the P301S model of tauopathy mitigates increased PHF1 levels and lipofuscin puncta and rescues memory deficits. eNeuro 3, ENEURO 0042. 10.1523/ENEURO.0042-16.2016 27257626 PMC4876487

[B107] Wang H. Wang R. Xu S. Lakshmana M. K. (2016b). Transcription factor EB is selectively reduced in the nuclear fractions of alzheimer's and amyotrophic lateral sclerosis brains. Neurosci. J. 2016, 4732837. 10.1155/2016/4732837 27433468 PMC4940567

[B108] Wang S. Yang F. Petyuk V. A. Shukla A. K. Monroe M. E. Gritsenko M. A. (2017). Quantitative proteomics identifies altered O-GlcNAcylation of structural, synaptic and memory-associated proteins in Alzheimer's disease. J. Pathol. 243, 78–88. 10.1002/path.4929 28657654 PMC5647145

[B109] Wang X. Li W. Marcus J. Pearson M. Song L. Smith K. (2020). MK-8719, a novel and selective O-GlcNAcase inhibitor that reduces the formation of pathological tau and ameliorates neurodegeneration in a mouse model of tauopathy. J. Pharmacol. Exp. Ther. 374, 252–263. 10.1124/jpet.120.266122 32493725

[B110] Weiss B. Allen G. E. Kloehn J. Abid K. Jaquier-Gubler P. Curran J. A. (2021). eIF4E3 forms an active eIF4F complex during stresses (eIF4FS) targeting mTOR and re-programs the translatome. Nucleic Acids Res. 49, 5159–5176. 10.1093/nar/gkab267 33893802 PMC8136781

[B111] Wesseling H. Mair W. Kumar M. Schlaffner C. N. Tang S. Beerepoot P. (2020). Tau PTM profiles identify patient heterogeneity and stages of alzheimer's disease. Cell 183, 1699–1713.e13. 10.1016/j.cell.2020.10.029 33188775 PMC8168922

[B112] Xu H. Jia C. Cheng C. Wu H. Cai H. Le W. (2022). Activation of autophagy attenuates motor deficits and extends lifespan in a *C. elegans* model of ALS. Free Radic. Biol. Med. 181, 52–61. 10.1016/j.freeradbiomed.2022.01.030 35114355 PMC8996503

[B113] Yakhine-Diop S. M. S. Niso-Santano M. Rodriguez-Arribas M. Gomez-Sanchez R. Martinez-Chacon G. Uribe-Carretero E. (2019). Impaired mitophagy and protein acetylation levels in fibroblasts from Parkinson's disease patients. Mol. Neurobiol. 56, 2466–2481. 10.1007/s12035-018-1206-6 30032424

[B114] Yim W. W. Mizushima N. (2020). Lysosome biology in autophagy. Cell Discov. 6, 6. 10.1038/s41421-020-0141-7 32047650 PMC7010707

[B115] Zavodszky E. Hegde R. S. (2019). Misfolded GPI-anchored proteins are escorted through the secretory pathway by ER-derived factors. Elife 8, e46740. 10.7554/eLife.46740 31094677 PMC6541436

[B116] Zhang P. Fan B. Yang P. Temirov J. Messing J. Kim H. J. (2019). Chronic optogenetic induction of stress granules is cytotoxic and reveals the evolution of ALS-FTD pathology. Elife 8, e39578. 10.7554/eLife.39578 30893049 PMC6426440

[B117] Zheng S. Clabough E. B. Sarkar S. Futter M. Rubinsztein D. C. Zeitlin S. O. (2010). Deletion of the huntingtin polyglutamine stretch enhances neuronal autophagy and longevity in mice. PLoS Genet. 6, e1000838. 10.1371/journal.pgen.1000838 20140187 PMC2816686

[B118] Zhou J. Wan J. Shu X. E. Mao Y. Liu X. M. Yuan X. (2018). N(6)-Methyladenosine guides mRNA alternative translation during integrated stress response. Mol. Cell 69, 636–647.e7. 10.1016/j.molcel.2018.01.019 29429926 PMC5816726

[B119] Zhuang X. X. Wang S. F. Tan Y. Song J. X. Zhu Z. Wang Z. Y. (2020). Pharmacological enhancement of TFEB-mediated autophagy alleviated neuronal death in oxidative stress-induced Parkinson's disease models. Cell Death Dis. 11, 128. 10.1038/s41419-020-2322-6 32071296 PMC7028954

